# The Genetic Diversity and Drug Resistance Patterns of HIV‐1 Pol Gene in East Africa

**DOI:** 10.1155/av/2801818

**Published:** 2025-10-17

**Authors:** Aneth Nzinyangwa Kavuraya, Teddy Mselle, Fulgence Ntangere Mpenda

**Affiliations:** ^1^ Department of Molecular Biology and Biotechnology, University of Dar es Salaam, P.O. Box 35179, Dar es Salaam, Tanzania, udsm.ac.tz; ^2^ Department of Biological and Pre-Clinical Studies, Muhimbili University of Health and Allied Sciences, P.O. Box 65001, Dar es Salaam, Tanzania, muchs.ac.tz; ^3^ Department of Biochemistry and Molecular Biology, Muhimbili University of Health and Allied Sciences, P.O. Box 65001, Dar es Salaam, Tanzania, muchs.ac.tz

**Keywords:** diversity, East Africa, HIV-1 subtypes, recombinants, Tanzania

## Abstract

Human immunodeficiency virus‐1 (HIV‐1) is among the most genetically diverse pathogens due to expeditious molecular evolution. The rapid change in HIV genomes intricates HIV transmission and progression and attributes HIV resistance to antiretroviral therapy (ART). In East Africa, as in other parts of the globe, HIV‐1 occurs in various subtypes, circulating recombinant form (CRF) and unique recombinant forms, with subtype A1 being the most predominant. Surveillance of HIV‐1 molecular diversity and drug resistance mutations (DRMs) is a linchpin for monitoring viral evolution and treatment efficiency. However, consolidated reports on the same are limited, and therefore, the pursuit of meta‐analysis was sought to analyze genetic diversity and drug resistance patterns of HIV‐1 pol gene and their geographical distributions in four East African countries (Kenya, Uganda, Tanzania, and Ethiopia). We retrieved 7614 HIV‐1 pol gene sequences, deposited between 2015 and 2025 from the Los Alamos HIV databases. The predominant HIV‐1 subtypes were A1 (40.2%), C (21.5%), and D (17.7%), with geographical variability. A notable frequency of inter‐subtype recombinant was observed with recombinants A1D (9.5%) and A1C (2.94%) being prevalent. Few CRFs (> 0.1%) were identified. DRM were present in 42.8% of the sequences, with the majority associated with NNRTIs (36.5%) and NRTIs (25.5%). The most frequently associated mutations were K103N and M184V. Although resistance to INSTI (3.7%) remained minimal, its presence warrants continued monitoring. A significant association between HIV‐1 subtypes and DRM prevalence was observed (*χ* ^2^ = 102.43, *p* < 0.0001), with subtypes showing varied resistance burdens. These findings underscore the variability in HIV‐1 genetic diversity across studied East African countries, highlighting the need for region‐specific interventions, to optimize HIV‐1 control in this region.

## 1. Introduction

Human immunodeficiency virus (HIV), the causative agent of acquired immunodeficiency syndrome (AIDS), is the most serious public health challenges. Taken as an example, globally, UNAIDS extrapolated that about 39.9 million people were living with HIV in 2023. Intriguing, in the same year, 1.3 million new infections and 630,000 HIV/AIDS attributed deaths were reported by Joint United Nations Programme on HIV/AIDS [1]. Although the distribution of HIV is skewed, Africa is particularly the most affected region accounting for about 65% of the global population of people living with HIV [2]. Likewise, the data from East and South Africa is alarming, in particular about 52% (20.8 million) of people living with HIV/AIDS reported in 2023 were from East and South Africa [3]. Sundry factors like high‐risk cultural practices, socioeconomic challenges, and limited access to healthcare have been portrayed to account for skewed burden of HIV in Africa, but the intrinsic factors like the HIV genetic variations and the temporal effects cannot be underestimated.

The HIV genome is approximately 9.7 kilobases in length and is composed of single‐stranded positive‐sense RNA [4]. There are two variants of HIV, which are HIV‐1 and HIV‐2. HIV‐1 is responsible for the majority of HIV infections worldwide and is categorized into four genetic groups: M (major or main), N (non‐M, non‐O) [5], O (outlier) [4], and P [6]. The groups of HIV‐1 have different geographical distributions, but have similar clinical symptoms [7]. Group M is the major group associated with the global HIV pandemic [8]. This group is further divided into 10 subtypes (A–D, F–H, and J–L) and 8 sub‐subtypes (A1, A2, A3, A4, A5, A6, F1, and F2) [9, 10]. Of interest, the subtypes/sub‐subtype may recombine to exist in recombinant forms and about 118 circulating recombinant forms (CRFs) have been reported. Nevertheless, some of the recombinants have not been documented, and therefore they are regarded as unique recombinant forms (URFs). The URFs are less prevalent and very unlikely to be detected. On the other hand, HIV‐2 is antithetical to HIV‐1 because HIV‐2 is less common and less virulent. HIV‐2 is mainly found in Western Africa with about 2 million people infected [11]. The present information reflects the remarkable genetic diversity of HIV‐1, which varies widely across regions and populations.

Being among the most genetically diverse pathogens, HIV‐1 undergoes constant molecular evolutions [12, 13]. This appreciable viral diversity within HIV‐1 is due to highly mutational escape and its adaptation to both immune activity and antiretroviral therapy (ART) [14]. The extensive genetic diversity of HIV‐1 is due to the following factors: (i) the high replication rate; (ii) the activity of reverse transcriptase (RT), which favors the accumulation of transcription errors that the enzyme is unable to correct since it lacks 3′ to 5′ exonuclease activity; (iii) the recombination events that may occur during virus replication; (iv) host selective immune pressure. [14–16]. This results in a dramatic change in genetic diversity and an increase in both intrahost variability (6%–19%) and interhost variability (2%–5%) [17]. As compared with other viral components, the Pol protein has higher mutation rate, and its role in viral survival has been highly studied.

The HIV‐1 pol gene encodes the three enzymes needed for viral replication: protease (PR), RT, and integrase (IN). These proteins have essential roles in the viral cycle and are the main targets of antiretroviral drugs (ARV) [16]. ARV drugs targeting the pol gene enzymes are divided into four groups: protease inhibitors (PIs), non‐nucleoside reverse transcriptase inhibitors (NNRTI), nucleoside reverse transcriptase inhibitors (NRTI), and recently introduced integrase transfer inhibitors (INSTIs) [18]. There are five PIs, ten RT inhibitors, and five INSTIs FDA approved and recommended in the U.S. Department of Health and Human Services (HHS) HIV/AIDS medical practice guidelines [19]. The identification of Pol mutations associated with ART through molecular detection and the use of online resistance algorithms has significantly enhanced resistance monitoring and ART selection for individuals with HIV [20–22].

Several studies have explored the genetic diversity and prevalence of HIV‐1 subtypes across East Africa (EA). In EA, the HIV subtype A has remained persistent over long epoch, and available reports highlights that more than half of HIV infections among the EA individuals are attributed to subtype A. However, other subtypes like subtypes C and D have relatively noticeable proportion of HIV infections in EA [17, 23]. In addition, few CRFs and URFs that contribute to the genetic complexity of HIV‐1 in the region are documented [24, 25]. HIV‐1 subtypes and recombinants may be associated with various phenotypes such as drug resistance evolution, disease progression, transmission patterns, and neuropsychological outcome.[26]. In terms of drug resistance, studies on East African population have consistently shown high resistance mutations rates to NNRTIs and NRTIs and low prevalence of resistance to PIs and INSTIs [27, 28]

Despite substantial global research on HIV diversity and drug resistance, comprehensive information in the region is limited and conjured the present study. This study analyzed HIV‐1 pol gene sequences from the Los Alamos HIV Database to characterize genetic diversity, subtypes, and drug resistance mutations (DRMs) across EA, with the findings providing insights into ART, enhance drug resistance surveillance, inform vaccine design, and strengthen regional HIV prevention and control strategies.

## 2. Materials and Methods

### 2.1. Study Overview

This study employed a meta‐analysis approach to evaluate the subtype distribution, diversity and drug resistance of the HIV‐1 pol gene across EA. Data on HIV‐1 pol gene sequences were retrieved from public database. Using appertaining software, the sequences were analyzed for assessment of DRMs in the pol gene enzymes (PR, RT, and IN).

### 2.2. Acquisition of HIV‐1 Pol Gene Sequences From Public Database

**Figure 1:**
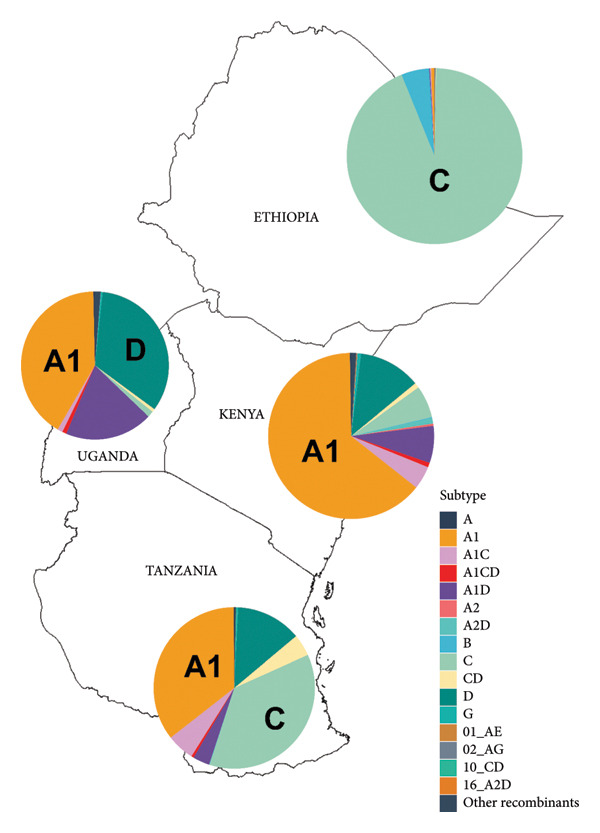
The East African map showing the geographic distribution of HIV‐1 pol gene subtypes in four countries based on the HIV‐1 pol gene sequences (2015–2025), which were retrieved from Los Alamos HIV sequence database (LANL). Pie charts show the percentage frequency of subtypes across Tanzania, Kenya, Uganda, and Ethiopia.

In June 2025, HIV‐1 pol gene sequences deposited between 2015 and 2025 from four East African countries were retrieved in FASTA format with subtyping information from the Los Alamos National Laboratory (LANL) HIV Database (https://www.hiv.lanl.gov). Duplicate accession numbers were removed, and only unique sequences were retained. Sequences with more than 3% IUPAC nucleotide ambiguity codes (e.g., M, R, Y) across the full pol gene were excluded from the dataset. The subtype result per country on a map in Figure 1 was created by using ArcMap software.

### 2.3. Assessment of DRMs in PR, RT, and IN Regions

Identification and classification of mutations associated with drug resistance in the PR, RT, and IN regions of the pol gene were carried out using the Stanford University HIV Drug Resistance Database (https://hivdb.stanford.edu/). The Stanford HIV Drug Resistance Database (HIVDB) is curated public database and essential resource for public health officials monitoring Acquired Drug Resistance and Transmitted Drug Resistance, for scientists developing new ARV drugs, and for HIV care providers managing patients with HIV. In addition, chi‐square (*χ* ^2^) tests were performed using Stata software version 16.0 to assess the relationship between HIV‐1 subtypes and drug resistance. Statistical significance was determined at a 95% confidence level within a marginal error of 0.05.

## 3. Results

### 3.1. The HIV‐1 Pol Gene Sequences Analyzed From EA in the Present Study

A total of 7724 HIV‐1 pol gene sequences were initially retrieved from LANL HIV sequence database, comprising sequence collected and deposited between 2015 and 2025. Following quality filtering which involved the removal of duplicate entries and sequences with high levels of ambiguous nucleotide characters, 110 sequences were excluded, resulting in 7614 sequences for downstream analysis. These included 5730 PR/RT, 734 RT, and 1594 IN sequences. The sequences were sourced from four EA countries: Kenya, Uganda, Tanzania, and Ethiopia. The distribution of HIV‐1 pol gene sequences by country is presented in Table 1.

**Table 1:** The HIV‐1 pol gene sequence from East Africa retrieved from public database by June 2025.

Country	Number of sequences	Pol gene enzyme	Sampling/collection date
Kenya	2040	RT‐69	2015–2022
		PR/RT‐1799	
		IN‐326	

Uganda	2673	RT‐546	2015–2022
		PR/RT‐1885	
		IN‐533	

Tanzania	1811	PR/RT‐1582	2015–2022
		IN‐229	

Ethiopia	1090	RT‐119	2015–2021
		PR/RT‐464	
		IN‐506	

*Note:* PR—protease, IN—integrase.

Abbreviation: RT, reverse transcriptase.

### 3.2. Genetic Diversity of HIV‐1 Pol Gene Subtypes Across East African Populations

Subtype with the greatest presentation among HIV‐1 pol gene sequences retrieved from four East African countries was subtype A1 accounting for 40.2% of all sequences. Of these, 42.7% originated from Kenya, 36.0% from Uganda, and 21.0% from Tanzania. Subtype C represented 24.2% of sequences, with majority from Ethiopia (55.02%), followed by Tanzania (35.9%). Subtype D accounted for 18.1% with the majority from Uganda (64.8%) followed by Kenya (18%) and Tanzania (17.3%), as shown in Table 2. Subtype A1 dominated in Kenya and Uganda, while subtype C was most common in Ethiopia and Tanzania. Inter‐subtype recombinants were notable, with subtype A1D comprising 9.5% (724 sequences) of the dataset, followed by A1C (2.9%) and CD (1.5%). CRFs such as 10_CD (0.07%) and 02_AG (0.05%) exhibited a low prevalence across the region. These findings highlight the dominance of subtypes A1, C, and D alongside a diverse array of recombinant forms in East African HIV‐1 populations.

**Table 2:** Geographic distribution of HIV‐1 subtypes in East African countries (2015–2025).

Subtype	Number of sequences per country				
	Uganda	Kenya	Tanzania	Ethiopia	Total
A1	1103	1308	642	7	3060
C	36	128	664	1020	1848
D	893	248	238	0	1379
A	9	5	2	0	16
B	0	3	1	56	60
G	11	14	3	0	28
A2	0	9	0	0	9
A1D	514	147	61	2	724
A1C	30	92	100	2	224
A2D	0	23	1	0	24
CD	18	20	79	1	118
A1CD	25	18	9	0	52
CRF16_A2D	0	2	0	0	2
CRF10_CD	0	0	5	0	5
CRF02_AG	1	2	0	1	4
CRF01_AE	0	1	0	0	1
Other recombinants	33	20	6	1	60

Total	2673	2040	1811	1090	7614

*Note:* Other recombinants: A1A2, A1A2D, A1B, A1G, DG, U, A1U, ADU, AU, and CF2.

The geographic distribution of HIV‐1 variants across the four countries, based on available pol sequences from the LANL, is illustrated in Figure 1.

### 3.3. Prevalence and Profiles of DRMs in HIV‐1 Pol Gene Sequences

**Figure 2:**
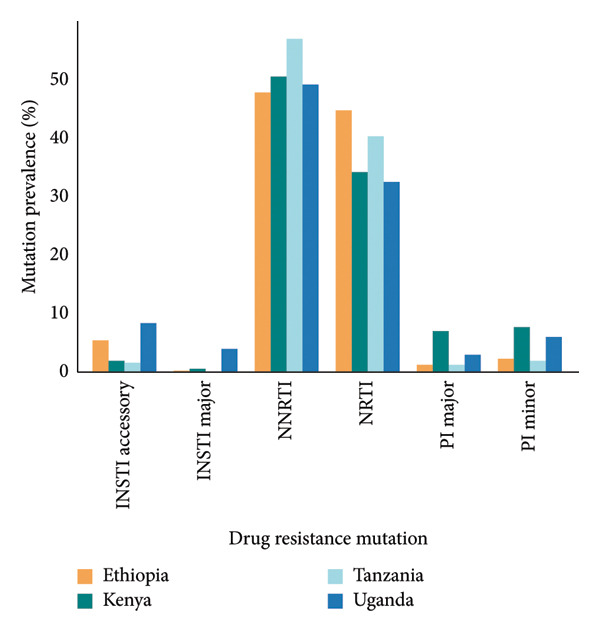
The distribution of drug resistance mutations associated with antiretroviral therapy (ART) in HIV‐1 pol gene sequences, comparing the prevalence of sequence with mutations across the four East African countries.

The overall prevalence of HIV‐1 pol gene sequences with at least one mutation across four East African countries was 42.8% (3266 sequences). The prevalence varied by country, with the highest observed in Kenya 51.2% (1045/2040), followed by Tanzania at 48.6% (880/1811), Uganda at 38.8% (1038/2673), and Ethiopia at 27.8% (303/1090). Mutations associated with NNRTI and NRTI were the most prevalent, accounting for 36.5% and 25.5% of all sequence analyzed, respectively. In contrast, sequences with PI‐ and INSTI‐associated mutations were less commonly identified in 6.2% and 3.7%, respectively. Notably, 280 out of the 3266 sequences (8.7%) harbored multiple mutations across more than one drug class region of the pol gene. The frequency distribution of sequences with at least one mutation by country is illustrated in Figure 2.

Out of 7614 reanalyzed sequences, 2779 (36.5%) harbored at least one NNRTI resistance–associated mutations, with a total of 5432 individual mutations observed. The most frequent NNRTI mutations were K103N, Y181C, and G190A. Variable levels of resistance to NNRTIs were identified across the dataset, with high‐level resistance most commonly observed against nevirapine (NVP) and efavirenz (EFV). Intermediate and low‐level resistance patterns were also noted, particularly for doravirine (DOR), etravirine (ETR), and rilpivirine (RPV) (Figure 3(a)).

Figure 3Levels of antiretroviral drug resistance in HIV‐1 pol gene sequences by drug class and mutation frequency. (a) NNRTI. DOR doravirine, EFV efavirenz, ETR etravirine, NVP nevirapine, RPV rilpivirine. (b) NRTI. ABC abacavir, ZDV zidovudine, D4T stavudine, DDI didanosine, FTC emtricitabine, 3TC lamivudine, TDF tenofovir disoproxil fumarate. (c) PI. r ritonavir‐boosted, ATV atazanavir, DRV darunavir, FPV fosamprenavir, IDV indinavir, LPV lopinavir, NFV nelfnavir, SQV saquinavir, TPV tipranavir. (d) INSTI. BIC bictegravir, CAB cabotegravir, DTG dolutegravir, EVG elvitegravir, RAL raltegravir.

**Figure:**
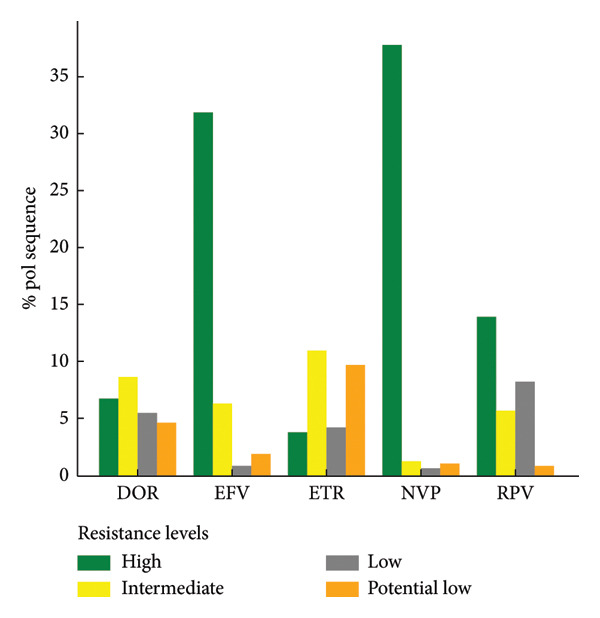
(a)

**Figure:**
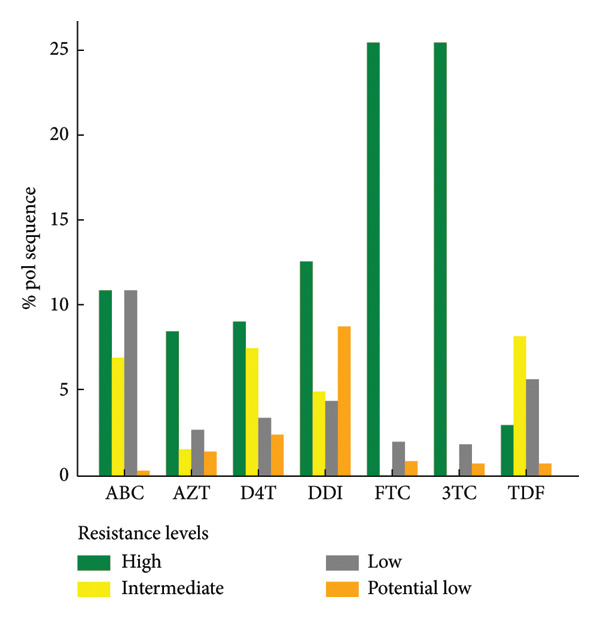
(b)

**Figure:**
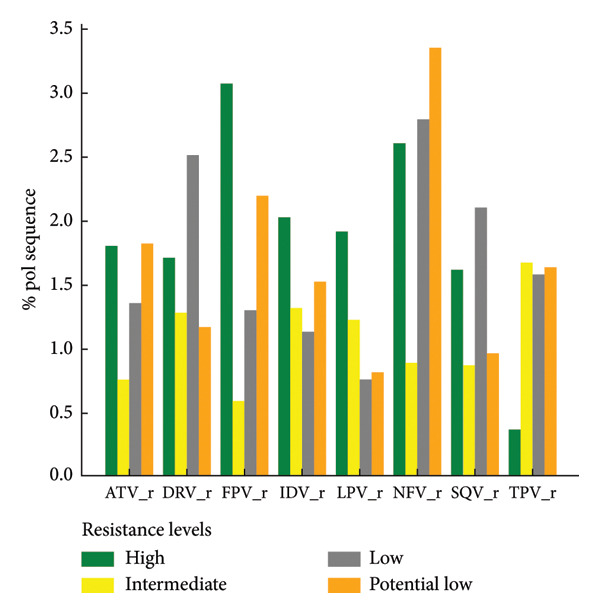
(c)

**Figure:**
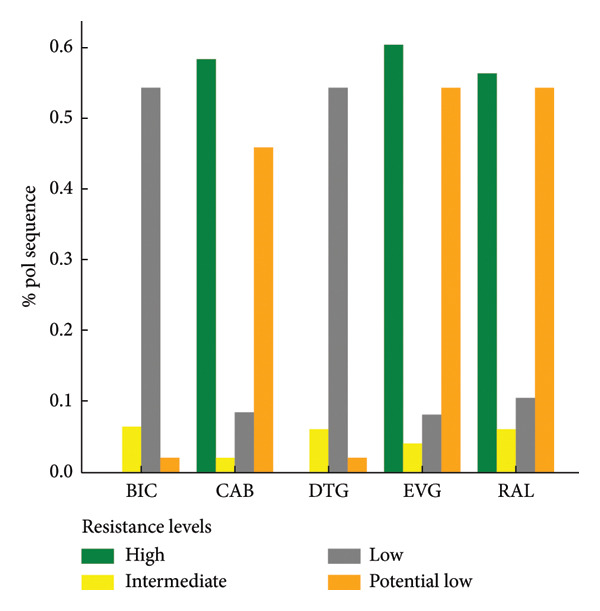
(d)

Among the studied sequences, 1941 (25.5%) sequences had at least one NRTI resistance–associated mutation, with a total of 5396 individual mutations observed. M184V, K56R, and D67N were among the most frequent NRTI mutations observed. A substantial proportion of sequences exhibited high‐level resistance to emtricitabine (FTC), lamivudine (3TC), and didanosine (DDI), as illustrated in Figure 3(b). Other levels of resistance to the mentioned NRTI drugs were also observed among the retrieved pol gene sequence, as illustrated in Figure 3(b).

PI resistance‐associated mutation was found in 469 (6.2%) sequences, comprising 451 major and 391 accessory mutations, giving a total of 842 individual PIs mutations. The most frequent PIs mutations including M46I, I54V, and L33F coffered high‐level resistance primarily to fosamprenavir/ritonavir (FPV/r), nelfinavir (NFV), and indinavir (IDV/r). A smaller proportion of sequences showed intermediate‐ and low‐level resistance to PIs drugs (Figure 3(c)). A total of 280 (3.7%) sequences were having INSTI resistance‐associated mutations, with a total of 322 individual mutations. The most common INSTI mutations were E138K and G140R, with few high‐ to intermediate‐level resistance observed in elvitegravir (EVG) and cabotegravir (CAB), while other resistance levels were rare (Figure 3(d)).

### 3.4. DRMs Among Different HIV‐1 Subtypes

A total of 7614 HIV‐1 sequences were analyzed, of which 3266 (42.9%) harbored DRMs. Subtype A1 was the most prevalent, comprising 40.2% (3060/7614) of all sequences and contributing the largest absolute number of resistance strains (1464/3266; 44.8%). Followed by subtypes, C (21.5%; 703/3266) and D (17.7%; 578/3266) were also frequent, with 703 and 578 resistant sequences, respectively, as shown in Table 3. Statistical results show that there was significant association between the HIV‐1 genotype and drug‐resistant mutations (*χ* ^2^ = 102.43; *p* ≤ 0.0001).

**Table 3:** Proportion of HIV‐1 sequences harboring drug‐resistant mutations (DRMs) by subtype.

HIV‐1 subtypes	No. of subtypes, *n* (%)	Sequence with DRM, *n* (%)	% with DRM within subtype
A1	3060 (40.2)	1464 (44.8)	47.8
C	1848 (24.3)	703 (21.5)	38
D	1379 (18.1)	578 (17.7)	41.2
A	16 (0.21)	4 (0.12)	25
B	60 (0.78)	7 (0.21)	11.6
G	28 (0.37)	12 (0.37)	42.8
A2	9 (0.12)	0 (0.0)	0.0
A1D	724 (9.5)	278 (8.5)	38.4
A1C	224 (2.94)	96 (2.9)	42.9
A2D	24 (0.32)	10 (0.31)	41.7
CD	118 (1.55)	58 (1.78)	49.2
A1CD	52 (0.68)	16 (0.49)	30.8
CRF16_A2D	2 (0.03)	2 (0.06)	100
CRF10_CD	5 (0.07)	2 (0.06)	40.0
CRF02_AG	4 (0.05)	1 (0.03)	25.0
CRF01_AE	1 (0.01)	0 (0.0)	0.0
Other recombinants	60 (0.78)	35 (1.07)	58.3

Total	7614	3266	

When resistance was evaluated relative to the number of sequences within each subtype, distinct different emerged. Subtype A1 showed a DRM prevalence of 47.8% (1464/3060), followed closely by subtype D at 41.9% (578/1379), while subtype C had a lower prevalence at 38.0% (703/1848). Less frequent subtypes and recombinants displayed high resistance rates despite their limited representation. For example, 42.9% (12/28) of subtype G and 58.3% (35/60) of “other recombinants” harbored DRMs. Several CRFs also demonstrated substantial resistance burdens, including CD (49.2%; 58/118), A1C (42.9%; 96/224), and A1D (38.4%; 278/724). Notably, CRF16_A2D sequences exhibited 100% resistance (2/2), although sample size was very small. In contrast, no resistance was detected in A2 (0/9) or CRF01_AE (0/1).

## 4. Discussion

Surveillance of HIV‐1 molecular diversity and DRMs is essential for monitoring viral evolution and informing treatment strategies. However, consolidated reports on this aspect have been limited in EA. This meta‐analysis sought to evaluate the genetic diversity and drug resistance patterns of HIV‐1 pol gene and their geographical distributions using publicly available data from 2015 to 2025 in the region.

These findings highlight variation in HIV‐1 diversity and geographical distribution across East African countries. Six subtypes, more than five inter‐subtype recombinants and few CRFs were identified. The observed diversity is generally comparable to other reports from Africa, with the exception of West Africa where diversity of CRFs surpasses that of pure subtypes [17]. The predominance of subtype A1 (40.2%) specifically in Kenya and Uganda aligns with previous reports identifying A1 as the dominant HIV‐1 subtype in EA, likely due to high transmissibility and historical spread [12, 17, 23]. The substantial presentation of subtype C (24.3%) particularly in Ethiopia and Tanzania, and subtype D (18.1%) in Uganda was noted. The current study confirmed the predominance of HIV‐1 subtype C in Ethiopia similar to previous study suggesting that subtype C accounts for 97% of the pandemic in the country [29].

The presence of inter‐subtype recombinants, notably A1D (9.5%), A1C (2.9%), and CD (1.5%), indicates ongoing recombination as a driver of HIV‐1 genetic diversity in EA and the possible introduction of other HIV‐1 subtypes from Neighboring countries [30]. Despite being low, the influx of other variants, including subtype B (0.78%), G (0.37%), and CRFs such as 01_AE, 02_AG, 10_CD, and 16_A2D (< 0.1% each), has been observed. The presence of other recombinant forms including URFs (0.78%) indicates sporadic and novel recombination events, expanding the genetic complexity of HIV‐1 in the region [17]. The occurrence of these other variants indicates the possible introduction of other HIV‐1 subtypes, and therefore preventive measures should always be there to prevent the possibility of further introductions of new subtypes in the region.

HIV‐1 drug resistance significantly challenges effective treatment, often leading to therapy failure and necessitating alternative therapeutic strategies [28, 31]. In this analysis, a total of 11,992 individual DRMs were identified in 3266 (42.8%) of the sequences. Majority of these mutations were associated with NNRTIs (36.5%) and NRTIs (25.5%), reflecting the prolonged reliance and associated resistance challenges posed by these drug classes since the inception of ART, as reported both globally and in regions [16, 27, 28, 32]. This highlights the significance of the current WHO guidelines to substitute NNRTIs in the fixed‐dose combinations with dolutegravir for HIV treatment (World Health organization, 2019). All East African countries have adopted the tenofovir, lamivudine, and dolutegravir (TLD) regimes as the first‐line therapy [33, 34].

High‐level resistance to nevirapine (NVP) and efavirenz (EFV) were observed largely among NNRTI due to key mutations such as K103N, Y181C, and G190A [35–37]. M184V was the most common NRTIs‐associated mutation conferring high‐level resistance to FTC and 3TC [22, 38, 39]. Thymidine analogue mutations (TAMs) such as T215Y, D67N, M41L, K219K/Q, and K70R were also identified among RT sequences, echoing previous reports from sub‐Saharan Africa [28, 34].

PI resistance mutations were less frequent (6.2%) suggesting that PIs may remain effective in this region. However, mutations such as M46I, I54V, and L33F indicate emerging resistance which can compromise the efficacy of certain PI‐based regimens. INSTIs resistance was rare, with only 280 (3.7%) sequences harboring mutations, suggesting that this class remains a viable option, particularly for salvage therapy. Notably, INSTI‐associated mutations such as E138K and G140R were detected. Although they generally have limited impact when presented alone, they have been associated with reduced susceptibility to Elvitegravir and Raltegravir in few cases, especially when occurring in combination with other resistance mutations particularly those at position 148 [40–42].

Studies reported that variation in DRM among HIV‐1 patients may be influenced by the genetic diversity of circulating subtypes and recombinants [43]. In this study, although subtype A1 contributed the highest absolute number of DRM‐harboring sequences (44.8%), this pattern is largely attributable to its predominance in the dataset (40.2%). When resistance was normalized by subtype frequency, other variants revealed comparable or even higher proportions of DRMs. Subtype G (42.8%) and the diverse pool of recombinants (up to 58.3% in other recombinants, and 49.2% in CD forms) showed disproportionate resistance burdens relative to their population size. These findings refine the reports that subtype A1 may be associated with DRMs in the EA region [38] by clarifying that this impact reflects its epidemiological dominance. Subtypes C and D also showed substantial frequencies of DRMs, with 703 (21.5%) and 578 (17.7%) mutated sequences, respectively.

The high resistance levels among recombinants were also notable, as recombination may accelerate the accumulation and dissemination of DRM patterns, thereby complicating both treatments monitoring and vaccine design [26, 44]. The complete resistance observed in CRF16_A2D, although based on very few sequences, highlights the need for close surveillance of rare forms that may escape detection in routine monitoring. Subtype C, the second most common subtype, showed a slightly lower proportion of DRMs (38.0%) compared with subtypes A1 (47.8%) and D (41.2%), which may reflect differences in drug exposure histories, treatment regimens, or intrinsic viral factors influencing mutational pathways. These subtype‐specific patterns could contribute to differential resistance profiles, as mutations accumulate at varying rates and positions across subtypes potentially impacting drug efficacy [32]. Therefore, the observed frequency of DRMs across subtypes underscores the importance of continued molecular surveillance and resistance monitoring to inform treatment strategies in the region.

The presented genetic diversity of HIV‐1 in EA is characterized by the predominance of pure subtypes and a notable presence of recombinant forms. The occurrence of DRMs particularly to NNRTIs and NRTIs, alongside a significant association between viral subtypes and resistance patterns, underscores the influence of genetic diversity on ART outcomes. Although the public database biases such as over‐representation of ART‐failure cases may inflate the observed resistance estimates, these findings emphasize the need for enhanced molecular surveillance tailored to regional viral diversity. Future studies should incorporate nonpublic datasets and real‐time sequencing to better capture evolving HIV‐1 dynamics, informing targeted ART strategies to strengthen HIV‐1 control across EA.

## 5. Conclusion

In this meta‐analysis, we evaluated the genetic diversity and geographical distribution of HIV‐1 pol gene in EA by analyzing publicly available sequences from 2015 to 2025. This was also the period where new HIV drug was introduced in the combination of first‐line ART regime (INSTI‐Dolutegravir in 2019). The continued dominance of subtype A1 and the emergence of recombinant forms reflect the dynamic and evolving nature of HIV‐1 in the region. The observed prevalence of resistance mutations highlights the need for region‐specific interventions, such as routine drug resistance testing to guide ART selection and enhanced surveillance of subtype‐specific resistance patterns, to optimize HIV‐1 control in EA. While resistance to newer drugs like INSTIs remains limited, its emergence signals the need for cautious use and sustained monitoring to preserve their effectiveness. However, given the limitations of public databases, particularly their incomplete representation of the broader HIV epidemic, future studies should incorporate nonpublic datasets and real‐time surveillance protocol. This will improve our understanding of HIV‐1 evolution and resistance trends and support the development of tailored interventions to control the epidemic more effectively in EA.

## 6. Limitations

A significant limitation of this study is its reliance on public databases, which capture only a fraction of available HIV‐1 sequence data and are subjected to sampling biases. Additionally, the exclusion of nonpublic datasets, such as those from large‐scale initiatives like the PANGEA program in Uganda, which have sequenced thousands of HIV‐1 samples, limits the generalizability of our findings. Globally, it is estimated that over 90% of HIV‐1 sequences are not deposited in public repositories due to patient privacy concerns and ethical restrictions. Moreover, the available sequence data in public repositories is inherently biased. Many of the sequences were generated as part of research studies that often target specific subpopulations, such as individuals experiencing ART failure or those participating in clinical trials. As a result, the dataset may be skewed toward viruses with DRMs, limiting the generalizability of the results to the broader HIV‐positive population, and also the inability to retrieve detailed metadata on treatment history, which prevent differentiation between transmitted and acquired DRMs which is an important distinction for interpreting resistance patterns.

## Conflicts of Interest

The authors declare no conflicts of interest.

## Funding

Funding for this study was provided by the High Education for Economic Transformation (HEET) project under MUHAS.

## Data Availability

The data used in this study were retrieved from publicly available database. The analyzed data are available from the corresponding author upon request.
